# 2-Amino-4,6-dimeth­oxy­pyrimidin-1-ium 2,2-dichloro­acetate

**DOI:** 10.1107/S1600536812021496

**Published:** 2012-05-26

**Authors:** Cui-Hua Lin, Nai-Sheng Liu

**Affiliations:** a Department of Chemistry and Chemical Engineering, Weifang University, Weifang 261061, People’s Republic of China; b Journal Editorial Department, Weifang University, Weifang 261061, People’s Republic of China

## Abstract

In the title salt, C_6_H_10_N_3_O_2_^+^·C_2_HCl_2_O_2_^−^, two cations and two anions are linked by N—H⋯O hydrogen bonds, forming chains along the *c* axis.

## Related literature

For the biological activity of heterocyclic compounds, see: Gilchrist (1998 ▶). For the bioactivity of pyrimidine derivatives, see: Xue *et al.* (1993 ▶). For a related structure, see: Hemamalini *et al.* (2005 ▶). For standard bond lengths, see: Allen *et al.* (1987 ▶).
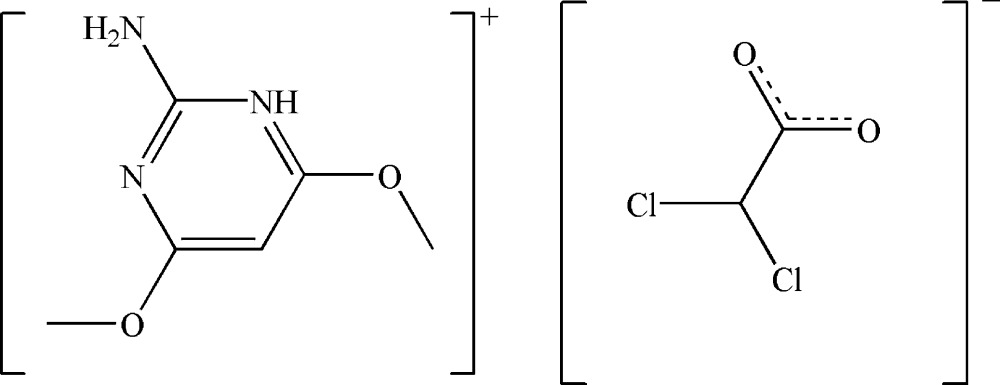


## Experimental

### 

#### Crystal data

C_6_H_10_N_3_O_2_^+^·C_2_HCl_2_O_2_^−^
*M* *_r_* = 284.10
Triclinic, 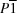

*a* = 6.8502 (14) Å
*b* = 8.6667 (17) Å
*c* = 11.255 (2) Å
α = 67.480 (1)°
β = 87.320 (2)°
γ = 85.970 (2)°
*V* = 615.6 (2) Å^3^
*Z* = 2
Mo *K*α radiation
μ = 0.53 mm^−1^
*T* = 293 K
0.45 × 0.43 × 0.35 mm

#### Data collection

Bruker SMART CCD diffractometer
Absorption correction: multi-scan (*SADABS*; Sheldrick, 1996 ▶) *T*_min_ = 0.795, *T*_max_ = 0.835
4710 measured reflections
2173 independent reflections
1806 reflections with *I* > 2σ(*I*)
*R*_int_ = 0.026

#### Refinement

*R*[*F* ^2^ > 2σ(*F* ^2^)] = 0.054
*wR*(*F* ^2^) = 0.200
*S* = 1.09
2173 reflections
156 parameters
H-atom parameters constrained
Δρ_max_ = 0.45 e Å^−3^
Δρ_min_ = −0.39 e Å^−3^

### 

Data collection: *SMART* (Bruker, 1997 ▶); cell refinement: *SAINT* (Bruker, 1997 ▶); data reduction: *SAINT*; program(s) used to solve structure: *SHELXS97* (Sheldrick, 2008 ▶); program(s) used to refine structure: *SHELXL97* (Sheldrick, 2008 ▶); molecular graphics: *SHELXTL* (Sheldrick, 2008 ▶) and *PLATON* (Spek, 2009 ▶); software used to prepare material for publication: *SHELXTL*.

## Supplementary Material

Crystal structure: contains datablock(s) I, global. DOI: 10.1107/S1600536812021496/lh5473sup1.cif

Structure factors: contains datablock(s) I. DOI: 10.1107/S1600536812021496/lh5473Isup2.hkl

Supplementary material file. DOI: 10.1107/S1600536812021496/lh5473Isup3.cml

Additional supplementary materials:  crystallographic information; 3D view; checkCIF report

## Figures and Tables

**Table 1:** Hydrogen-bond geometry (Å, °)

*D*—H⋯*A*	*D*—H	H⋯*A*	*D*⋯*A*	*D*—H⋯*A*
N3—H3*B*⋯O3^i^	0.86	1.97	2.822 (3)	173
N3—H3*A*⋯O3^ii^	0.86	2.07	2.848 (3)	149
N2—H2⋯O4^i^	0.86	1.85	2.692 (3)	168

Symmetry codes: (i) 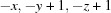; (ii) 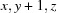.

**Fig. 1.**
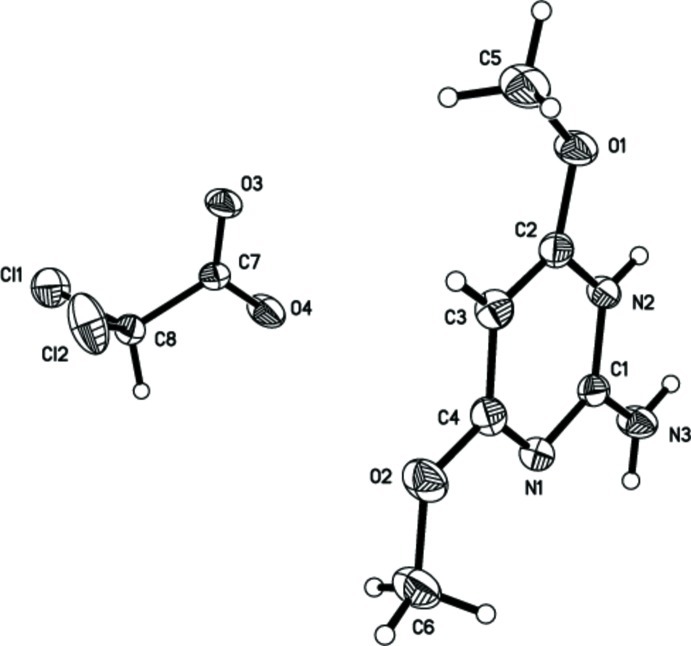
The structure of the title compound showing 30% probability displacement ellipsoids.

**Fig. 2.**
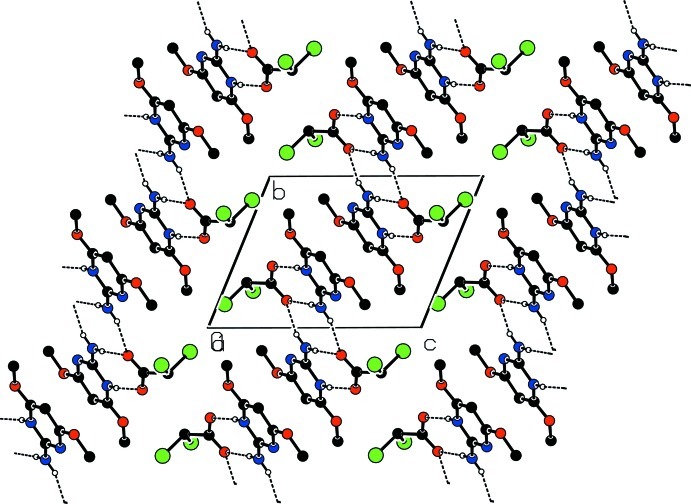
Part of the crystal structure with hydrogen bonds shown as dashed lines.

## supplementary crystallographic information

### Comment

Five and six-membered heterocyclic compounds are important constituents that often exist in biologically active natural products and synthetic compounds of medicinal interest (Gilchrist, 1998). As useful precursors to potentially bioactive pyrimidine derivatives, methylpyrimidine has attracted considerable attention for many years (Xue *et al.*, 1993). In recent years, new complexes of pyrimidine have been synthesized (Hemamalini *et al.*, 2005). Herein we report herein the crystal structure of the title compound (I).

The molecular structure of (I) is shown in Fig. 1. There is one cation and one anion in the asymmetric unit of (I). All bond lengths are within the normal ranges (Allen *et al.*, 1987). In the crystal, two cations and two anions are linked by intermolecular N—H···O hydrogen bonds to form centrosymmetric four component aggregates.

### Experimental

A mixture of 2-amino-4,6-dichloropyrimidine (0.1 mol) and sodium methoxide (0.1 mol) was stirred with methanol (30 ml) for 3 h to afford 2-Amino-4,6-dimethoxypyrimidine (yield 85%). The title compound was crystallized from an aqueous mixture containing 2-Amino-4,6-dimethoxypyrimidine and dichloroacetate in a 1:1 stoichiometric ratio at room temperature by the slow evaporation technique.

### Refinement

H atoms bonded to C atoms were fixed geometrically and and included in a riding-model approximation with C—H = 0.93–0.98 Å and N—H = 0.86Å with *U*_iso_(H)=1.2–1.5*U*_eq_(C).

### Crystal data

**Table:**

C_6_H_10_N_3_O_2_^+^·C_2_HCl_2_O_2_^−^	*Z* = 2
*M**_r_* = 284.10	*F*(000) = 292
Triclinic, *P*1	*D*_x_ = 1.533 Mg m^−^^3^
Hall symbol: -P 1	Mo *K*α radiation, λ = 0.71073 Å
*a* = 6.8502 (14) Å	Cell parameters from 3794 reflections
*b* = 8.6667 (17) Å	θ = 3.5–27.5°
*c* = 11.255 (2) Å	µ = 0.53 mm^−^^1^
α = 67.480 (1)°	*T* = 293 K
β = 87.320 (2)°	Block, colorless
γ = 85.970 (2)°	0.45 × 0.43 × 0.35 mm
*V* = 615.6 (2) Å^3^	

### Data collection

**Table:**

Bruker SMART CCD diffractometer	2173 independent reflections
Radiation source: fine-focus sealed tube	1806 reflections with *I* > 2σ(*I*)
Graphite monochromator	*R*_int_ = 0.026
φ and ω scans	θ_max_ = 25.0°, θ_min_ = 3.5°
Absorption correction: multi-scan (*SADABS*; Sheldrick, 1996)	*h* = −8→7
*T*_min_ = 0.795, *T*_max_ = 0.835	*k* = −10→10
4710 measured reflections	*l* = −13→13

### Refinement

**Table:**

Refinement on *F*^2^	Primary atom site location: structure-invariant direct methods
Least-squares matrix: full	Secondary atom site location: difference Fourier map
*R*[*F*^2^ > 2σ(*F*^2^)] = 0.054	Hydrogen site location: inferred from neighbouring sites
*wR*(*F*^2^) = 0.200	H-atom parameters constrained
*S* = 1.09	*w* = 1/[σ^2^(*F*_o_^2^) + (0.1265*P*)^2^ + 0.3628*P*] where *P* = (*F*_o_^2^ + 2*F*_c_^2^)/3
2173 reflections	(Δ/σ)_max_ < 0.001
156 parameters	Δρ_max_ = 0.45 e Å^−^^3^
0 restraints	Δρ_min_ = −0.39 e Å^−^^3^

### Special details

**Table:**

Geometry. All e.s.d.'s (except the e.s.d. in the dihedral angle between two l.s. planes) are estimated using the full covariance matrix. The cell e.s.d.'s are taken into account individually in the estimation of e.s.d.'s in distances, angles and torsion angles; correlations between e.s.d.'s in cell parameters are only used when they are defined by crystal symmetry. An approximate (isotropic) treatment of cell e.s.d.'s is used for estimating e.s.d.'s involving l.s. planes.
Refinement. Refinement of *F*^2^ against ALL reflections. The weighted *R*-factor *wR* and goodness of fit *S* are based on *F*^2^, conventional *R*-factors *R* are based on *F*, with *F* set to zero for negative *F*^2^. The threshold expression of *F*^2^ > σ(*F*^2^) is used only for calculating *R*-factors(gt) *etc*. and is not relevant to the choice of reflections for refinement. *R*-factors based on *F*^2^ are statistically about twice as large as those based on *F*, and *R*- factors based on ALL data will be even larger.

### Fractional atomic coordinates and isotropic or equivalent isotropic displacement parameters (Å^2^)

**Table:**

	*x*	*y*	*z*	*U*_iso_*/*U*_eq_	
Cl1	0.11057 (16)	0.15674 (13)	0.03526 (9)	0.0641 (4)	
Cl2	0.44050 (14)	0.24515 (19)	0.14101 (11)	0.0804 (5)	
N1	0.5368 (4)	0.7901 (3)	0.4560 (2)	0.0365 (6)	
N2	0.3706 (3)	0.6207 (3)	0.6442 (2)	0.0336 (6)	
H2	0.2667	0.6043	0.6924	0.040*	
N3	0.2252 (4)	0.8660 (3)	0.5053 (3)	0.0450 (7)	
H3A	0.2264	0.9542	0.4356	0.054*	
H3B	0.1235	0.8474	0.5555	0.054*	
O1	0.4900 (3)	0.3802 (3)	0.7880 (2)	0.0487 (6)	
O2	0.8446 (3)	0.7020 (3)	0.4156 (2)	0.0530 (7)	
O3	0.0938 (4)	0.1810 (3)	0.3189 (2)	0.0511 (7)	
O4	−0.0703 (4)	0.4064 (3)	0.1873 (2)	0.0653 (8)	
C1	0.3790 (4)	0.7598 (3)	0.5350 (3)	0.0332 (6)	
C2	0.5248 (4)	0.5073 (4)	0.6779 (3)	0.0367 (7)	
C3	0.6887 (4)	0.5305 (4)	0.6026 (3)	0.0389 (7)	
H3	0.7962	0.4540	0.6232	0.047*	
C4	0.6855 (4)	0.6780 (4)	0.4910 (3)	0.0384 (7)	
C5	0.6408 (6)	0.2493 (5)	0.8325 (4)	0.0651 (11)	
H5A	0.6728	0.2056	0.7670	0.098*	
H5B	0.5956	0.1614	0.9089	0.098*	
H5C	0.7551	0.2931	0.8514	0.098*	
C6	0.8456 (6)	0.8491 (5)	0.3002 (4)	0.0614 (10)	
H6A	0.7688	0.8339	0.2367	0.092*	
H6B	0.9777	0.8690	0.2682	0.092*	
H6C	0.7910	0.9432	0.3181	0.092*	
C7	0.0616 (4)	0.2935 (4)	0.2130 (3)	0.0378 (7)	
C8	0.1932 (4)	0.2995 (4)	0.0968 (3)	0.0394 (7)	
H8	0.1832	0.4127	0.0302	0.047*	

### Atomic displacement parameters (Å^2^)

**Table:**

	*U*^11^	*U*^22^	*U*^33^	*U*^12^	*U*^13^	*U*^23^
Cl1	0.0840 (8)	0.0668 (7)	0.0495 (6)	−0.0127 (5)	−0.0068 (5)	−0.0292 (5)
Cl2	0.0425 (6)	0.1419 (12)	0.0752 (7)	0.0083 (6)	−0.0034 (5)	−0.0639 (8)
N1	0.0382 (13)	0.0356 (13)	0.0345 (13)	−0.0043 (10)	0.0020 (10)	−0.0121 (10)
N2	0.0329 (12)	0.0350 (13)	0.0292 (12)	0.0017 (10)	0.0014 (9)	−0.0089 (10)
N3	0.0435 (14)	0.0386 (14)	0.0392 (14)	0.0085 (11)	0.0022 (11)	−0.0016 (11)
O1	0.0530 (13)	0.0411 (12)	0.0371 (12)	0.0138 (10)	0.0023 (10)	−0.0015 (9)
O2	0.0392 (12)	0.0607 (16)	0.0543 (14)	−0.0042 (11)	0.0145 (10)	−0.0181 (12)
O3	0.0588 (15)	0.0438 (13)	0.0337 (12)	0.0107 (10)	0.0032 (10)	0.0012 (10)
O4	0.0660 (16)	0.0588 (16)	0.0430 (13)	0.0296 (13)	0.0131 (12)	0.0046 (11)
C1	0.0372 (15)	0.0319 (14)	0.0311 (14)	−0.0031 (11)	−0.0025 (11)	−0.0124 (11)
C2	0.0424 (16)	0.0340 (15)	0.0334 (14)	0.0028 (12)	−0.0045 (12)	−0.0128 (12)
C3	0.0330 (15)	0.0410 (16)	0.0416 (16)	0.0042 (12)	−0.0039 (12)	−0.0152 (13)
C4	0.0348 (15)	0.0445 (17)	0.0396 (16)	−0.0071 (13)	0.0019 (12)	−0.0197 (13)
C5	0.070 (2)	0.059 (2)	0.0451 (19)	0.0295 (19)	−0.0043 (18)	−0.0025 (17)
C6	0.057 (2)	0.068 (3)	0.052 (2)	−0.0141 (18)	0.0182 (17)	−0.0157 (18)
C7	0.0431 (16)	0.0315 (15)	0.0340 (15)	0.0008 (12)	0.0002 (12)	−0.0076 (12)
C8	0.0445 (17)	0.0375 (16)	0.0339 (15)	0.0009 (12)	0.0003 (12)	−0.0116 (12)

### Geometric parameters (Å, º)

**Table:**

Cl1—C8	1.767 (3)	O3—C7	1.234 (4)
Cl2—C8	1.768 (3)	O4—C7	1.241 (4)
N1—C4	1.320 (4)	C2—C3	1.353 (4)
N1—C1	1.342 (4)	C3—C4	1.408 (4)
N2—C2	1.354 (4)	C3—H3	0.9300
N2—C1	1.355 (4)	C5—H5A	0.9600
N2—H2	0.8600	C5—H5B	0.9600
N3—C1	1.315 (4)	C5—H5C	0.9600
N3—H3A	0.8600	C6—H6A	0.9600
N3—H3B	0.8600	C6—H6B	0.9600
O1—C2	1.330 (4)	C6—H6C	0.9600
O1—C5	1.433 (4)	C7—C8	1.539 (4)
O2—C4	1.327 (4)	C8—H8	0.9800
O2—C6	1.430 (5)		
			
C4—N1—C1	116.5 (2)	O1—C5—H5A	109.5
C2—N2—C1	120.4 (2)	O1—C5—H5B	109.5
C2—N2—H2	119.8	H5A—C5—H5B	109.5
C1—N2—H2	119.8	O1—C5—H5C	109.5
C1—N3—H3A	120.0	H5A—C5—H5C	109.5
C1—N3—H3B	120.0	H5B—C5—H5C	109.5
H3A—N3—H3B	120.0	O2—C6—H6A	109.5
C2—O1—C5	117.1 (3)	O2—C6—H6B	109.5
C4—O2—C6	117.9 (3)	H6A—C6—H6B	109.5
N3—C1—N1	119.5 (3)	O2—C6—H6C	109.5
N3—C1—N2	118.4 (3)	H6A—C6—H6C	109.5
N1—C1—N2	122.1 (3)	H6B—C6—H6C	109.5
O1—C2—C3	127.8 (3)	O3—C7—O4	126.9 (3)
O1—C2—N2	111.7 (3)	O3—C7—C8	119.0 (3)
C3—C2—N2	120.5 (3)	O4—C7—C8	114.1 (3)
C2—C3—C4	115.5 (3)	C7—C8—Cl1	108.5 (2)
C2—C3—H3	122.2	C7—C8—Cl2	111.4 (2)
C4—C3—H3	122.2	Cl1—C8—Cl2	109.21 (17)
N1—C4—O2	118.7 (3)	C7—C8—H8	109.3
N1—C4—C3	125.0 (3)	Cl1—C8—H8	109.3
O2—C4—C3	116.3 (3)	Cl2—C8—H8	109.3
			
C4—N1—C1—N3	−179.5 (3)	C1—N1—C4—O2	179.6 (3)
C4—N1—C1—N2	−0.8 (4)	C1—N1—C4—C3	0.9 (4)
C2—N2—C1—N3	179.4 (3)	C6—O2—C4—N1	0.1 (4)
C2—N2—C1—N1	0.7 (4)	C6—O2—C4—C3	179.0 (3)
C5—O1—C2—C3	−1.4 (5)	C2—C3—C4—N1	−0.7 (5)
C5—O1—C2—N2	178.5 (3)	C2—C3—C4—O2	−179.5 (3)
C1—N2—C2—O1	179.6 (3)	O3—C7—C8—Cl1	81.6 (3)
C1—N2—C2—C3	−0.5 (4)	O4—C7—C8—Cl1	−97.9 (3)
O1—C2—C3—C4	−179.6 (3)	O3—C7—C8—Cl2	−38.6 (4)
N2—C2—C3—C4	0.5 (4)	O4—C7—C8—Cl2	141.9 (3)

### Hydrogen-bond geometry (Å, º)

**Table:**

*D*—H···*A*	*D*—H	H···*A*	*D*···*A*	*D*—H···*A*
N3—H3*B*···O3^i^	0.86	1.97	2.822 (3)	173
N3—H3*A*···O3^ii^	0.86	2.07	2.848 (3)	149
N2—H2···O4^i^	0.86	1.85	2.692 (3)	168

Symmetry codes: (i) −*x*, −*y*+1, −*z*+1; (ii) *x*, *y*+1, *z*.
